# Viral G Protein–Coupled Receptors Encoded by β- and γ-Herpesviruses

**DOI:** 10.1146/annurev-virology-100220-113942

**Published:** 2022-06-07

**Authors:** Mette M. Rosenkilde, Naotaka Tsutsumi, Julius M. Knerr, Dagmar F. Kildedal, K. Christopher Garcia

**Affiliations:** 1 Department of Biomedical Sciences, University of Copenhagen, Copenhagen, Denmark; 2 Graduate School of Medicine, Dentistry, and Pharmaceutical Sciences, Okayama University, Okayama, Japan; 3 Synklino Aps, Charlottenlund, Denmark; 4 Departments of Molecular and Cellular Physiology, and Structural Biology, and Howard Hughes Medical Institute, Stanford University School of Medicine, Stanford, California, USA

**Keywords:** herpesvirus, chemokine receptor, broad-spectrum ligand binding, G protein signaling, GPCR structure

## Abstract

Herpesviruses are ancient large DNA viruses that have exploited gene capture as part of their strategy to escape immune surveillance, promote virus spreading, or reprogram host cells to benefit their survival. Most acquired genes are transmembrane proteins and cytokines, such as viral G protein–coupled receptors (vGPCRs), chemokines, and chemokine-binding proteins. This review focuses on the vGPCRs encoded by the human β- and γ-herpesviruses. These include receptors from human cytomegalovirus, which encodes four vGPCRs: US27, US28, UL33, and UL78; human herpesvirus 6 and 7 with two receptors: U12 and U51; Epstein-Barr virus with one: BILF1; and Kaposi’s sarcoma-associated herpesvirus with one: open reading frame 74, ORF74. We discuss ligand binding, signaling, and structures of the vGPCRs in light of robust differences from endogenous receptors. Finally, we briefly discuss the therapeutic targeting of vGPCRs as future treatment of acute and chronic herpesvirus infections.

## 1. INTRODUCTION

### 1.1. Herpesviruses Exploit G Protein–Coupled Receptors

**Figure 1:**
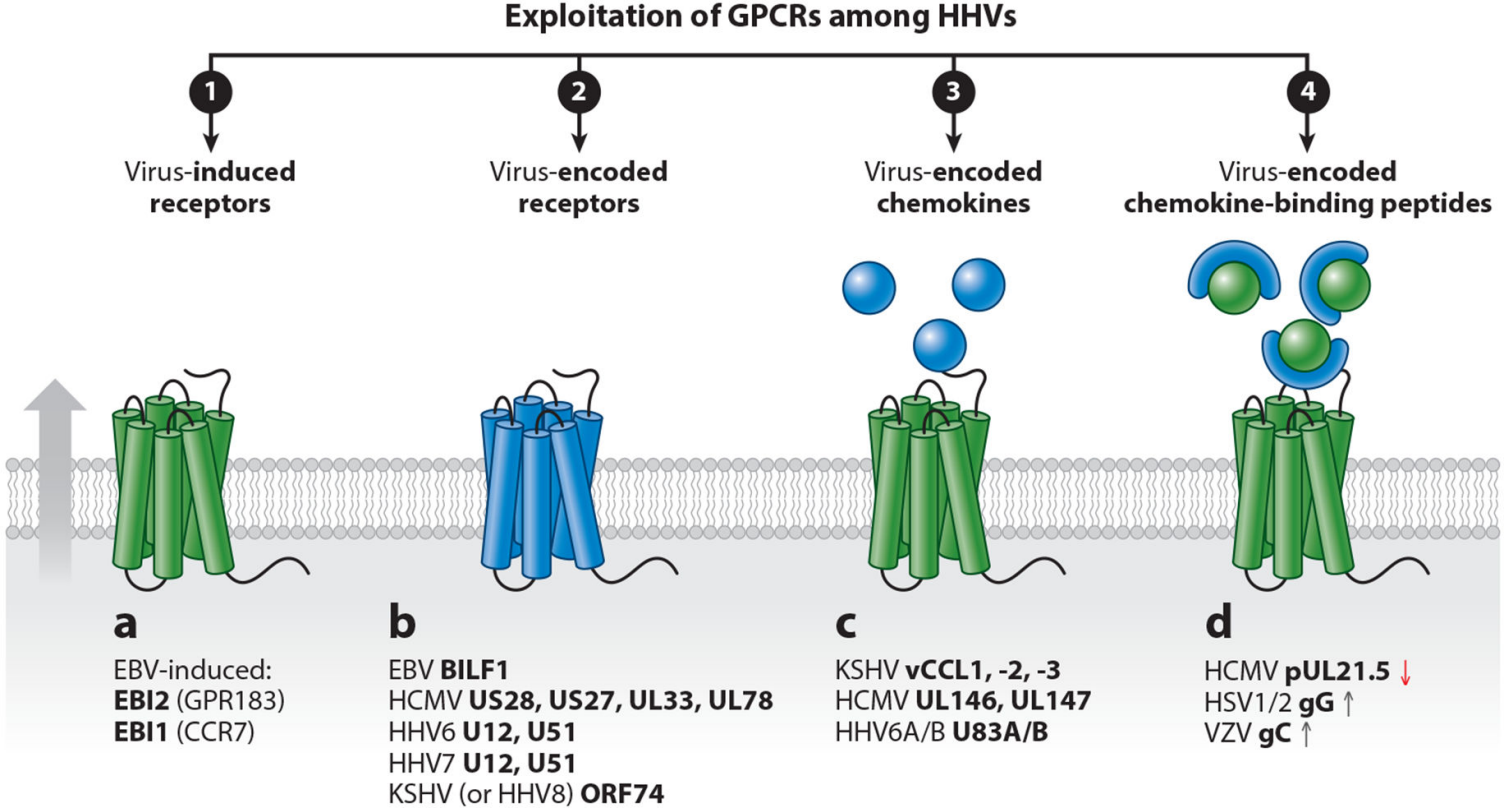
Four principles involving GPCRs and their ligands by which viruses regulate their host. Viruses (*a*) induce or reduce expression of endogenous receptors, (*b*) express vGPCRs, (*c*) express chemokines, or (*d*) express chemokine-binding proteins to impact the host immune system. Abbreviations: EBI, Epstein-Barr virus–induced gene; EBV, Epstein-Barr virus; GPCR, G protein–coupled receptor; HCMV, human cytomegalovirus; HHV, human herpesvirus; HSV, herpes simplex virus; KSHV, Kaposi’s sarcoma-associated herpesvirus; vGPCR, viral G protein–coupled receptor; VZV, varicella-zoster virus. Figure adapted from images created with BioRender.com.

Herpesviruses are large double-stranded DNA viruses that establish lifelong, latent infections with reactivation occurring, for instance, during periods of immune suppression. A striking feature of the herpesviruses is that they have exploited gene capture within the family of G protein–coupled receptors (GPCRs) and their ligands via an ancient act of molecular piracy. Through evolution, these captured genes have been optimized to serve a specific phenotype to benefit the viral life cycle, by either exploitation or subversion of the immune system by molecular mimicry (Figure 1). The viral GPCRs (vGPCRs) often show homology to endogenous chemokine receptors, and the secreted proteins include endogenous chemokine orthologs. Moreover, chemokine-binding proteins are found in several herpesviruses, with no resemblance to human proteins. As another way of controlling the host immune system, herpesviruses regulate the expression of leukocyte-expressed receptors, exemplified by the high induction of the two receptors, Epstein-Barr virus (EBV)–induced gene 1 and 2 (EBI1 and EBI2) (1). These are both Gi-coupled chemotactic receptors (2, 3) and have been deorphanized as CCR7 for EBI1 (activated by CCL19 and CCL21) and GPR183 for EBI2 (activated by oxysterols) (4). Other chemokine receptors and cytokines are also regulated upon herpesvirus cell entry, but as host genes, these are not the focus of the current review (5).

Mammalian herpesviruses comprise three major subfamilies, the α-, β-, and γ-herpesviruses, based on overall genome structure and subfamily-specific features, including several conserved gene families (6, 7) (Table 1). Of these, the human α-herpesviruses are composed of herpes simplex virus 1 and 2 (HSV1 and HSV2) and varicella-zoster virus (VZV or HHV3). The human β-herpesviruses comprise human cytomegalovirus (HCMV or HHV5) and human herpesvirus 6 and 7 (HHV6 and HHV7). The human γ-herpesviruses are divided into the lymphocryptovirus (γ1) with one member, EBV or HHV4, and the rhadinoviruses (γ2) with one member, the Kaposi’s sarcoma-associated herpesvirus (KSHV or HHV8).

### 1.2. Endogenous G Protein–Coupled Receptors

With approximately 800 members, GPCRs represent the largest protein family encoded by the human genome (8). They share a central domain with seven transmembrane (TM) helices connected by extracellular and intracellular loops, hence known as 7TM receptors. They transduce extracellular signals into the intracellular region, thereby controlling key physiological and pathophysiological effects within major areas such as the cardiovascular system, the musculoskeletal system, and the immune system as well as pain perception (9). This process occurs through interactions with a large variety of extracellular molecules and forces, spanning from photons, ions, peptides, carbohydrates, and lipids to proteins, inducing conformational changes in the receptor to initiate signal transduction cascades (9, 10). Intracellularly, the receptors couple with G proteins (four major classes: Gαs, Gαi, Gαq, and Gα12/13) or arrestins (arrestin 1–4) to mediate signaling cascades. The human GPCR superfamily is divided into class A (rhodopsin-like), class B1 (secretin), class B2 (adhesion receptors), class C (glutamate receptors), class F (Frizzled receptors), and class T (taste 2 receptors).

In recent years, a plethora of GPCR structures have been revealed, initially using crystallography but later cryogenic electron microscopy (cryo-EM), either with or without complexes with ligands or intracellular proteins (8). The structures of several vGPCRs have also been solved, revealing marked differences from their closest human class A receptor homologs.

### 1.3. Chemokines and Their Receptors

**Figure 2:**
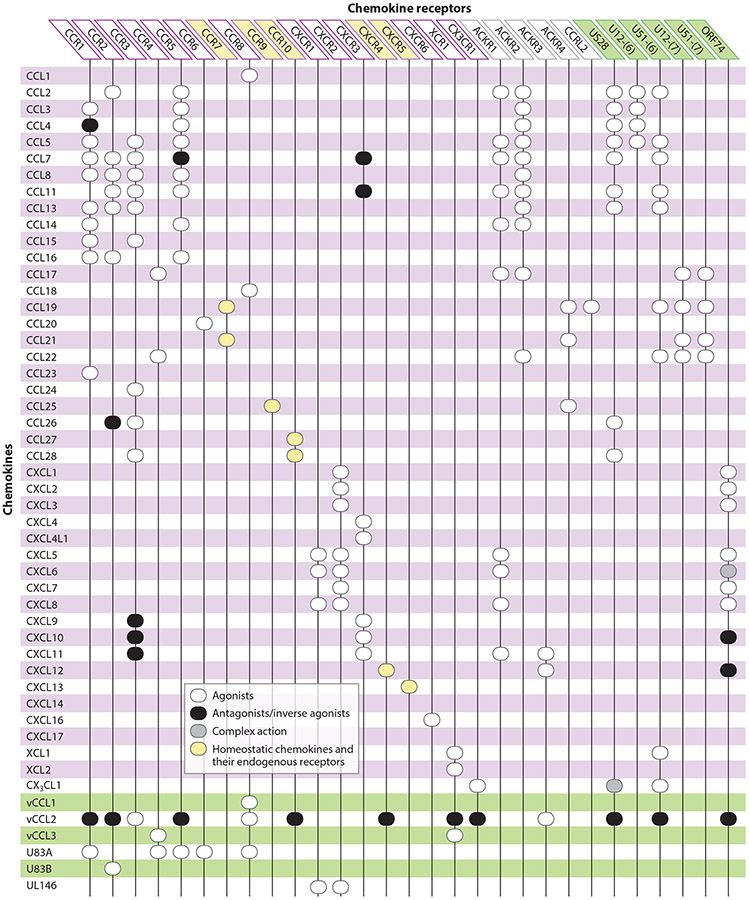
Overview of the complex interactions between chemokines and their chemokine receptors. Viral G protein–coupled receptors and the viral chemokines are highlighted in green. Figure adapted from References 11, 21, 23, and 152.

Chemokines (or chemotactic cytokines) constitute a family of leukocyte chemoattractant proteins that exert their effect through interaction with chemokine receptors belonging to class A GPCRs (11). Besides playing a leading role in directional migration, chemokines also regulate activation and differentiation of all subsets of leukocytes and are involved in regulatory processes outside the hematopoietic compartments, such as angiogenesis, organ formation, and carcinogenesis (11). The chemokines are divided into two major families, CC and CXC chemokines, and two minor families, XC and CX3C chemokines. This division is based on the primary sequence of their N termini, where the presence or absence of an amino acid between the first two of four conserved cysteines defines the two major families: CCL1 through −28 and CXCL1 through −17. In contrast to these two large groups, the two minor groups are constituted by XCL1 and −2, which lack the first conserved cysteine, and CX3CL1, which has three amino acids between the first two cysteines (Figure 2). The CXC chemokines are further divided into two groups based on the presence of an ELR (Glu-Leu-Arg) motif prior to the CXC motif. ELR CXC chemokines (CXCL1–3, 5–8) mainly attract neutrophils, whereas non-ELR CXC chemokines (CXCL4, CXCL9–17) mainly act on lymphocytes and nonhematopoietic cells. Moreover, angiogenesis is strictly controlled by these as ELR CXC chemokines are angiogenic, while non-ELR CXC chemokines are angiostatic or angio-modulatory (12).

The corresponding chemokine receptors are named from their primary ligand-targeting profiles as CCR1–10, CXCR1–6, XCR1, and CX3CR1. Endogenous chemokine receptors primarily signal through G proteins belonging to the Gαi subgroup (11, 13). Moreover, they recruit arrestins that are important regulators of receptor signaling and expression, empowering chemokine receptors to sense a chemokine gradient for ligand-directed migration (11). The endogenous chemokine receptors and their cognate chemokine agonists are functionally classified into three groups, homeostatic, inflammatory, and dual inflammatory/homeostatic subtypes, according to whether they are used for immune system development and basal leukocyte trafficking (homeostatic), emergency trafficking of leukocytes to sites of infection or tissue injury (inflammatory), or both (inflammatory/homeostatic) (11). Of these, the inflammatory chemokines are the most abundant (Figure 2).

An important feature of the human chemokine-chemokine receptor system is that it includes both chemokines that are monogamously paired to specific receptors and chemokines that are highly promiscuous and activate several receptors (Figure 2). For example, CCL1 binds CCR8, while CCL5 binds CCR1, −3, and −5, in addition to two atypical chemokine receptors (ACKR1 and −2) and three vGPCRs (US28 and U12 as well as U51 from HHV6). Similarly, certain receptors, such as CCR5, bind multiple chemokines. Among the chemokines for CCR5, the macrophage inflammatory proteins (MIPs) (CCL3–5) were identified first, but later the monocyte chemoattractant proteins (MCPs) (CCL2, −7, and −8), traditionally categorized as the cognate CCR2 agonists, were described as CCR5 agonists (11). The complexity is further exemplified by an overlap in most of the CCR5 chemokines to also bind CCR1 and an overlap in cellular expression of CCR1, −2, and −5 (11, 14). A similar pattern is found within the inflammatory CXC chemokines and their receptors, where CXCR2 is targeted by a multitude of ELR CXC chemokines, particularly in controlling neutrophil migration. This high degree of promiscuity probably reflects the evolutionary pressure on the immune system exerted by different types of pathogens (14). In contrast, a much higher degree of monogamy is found within the homeostatic chemokines and their receptors, where one or two exclusive cognate chemokines exist for each receptor (Figure 2).

### 1.4. Atypical Chemokine Receptors

The five members of this family are ACKR1–4 and CCRL2. ACKR1–4 share many features with vGPCRs in their broad chemokine-binding profiles crossing the CC- and CXC-chemokine boundaries (Figure 2), constitutive internalization, strong arrestin coupling, and regulation of endogenous chemokine receptor functions by heterodimerization (15). However, they lack G protein coupling, both upon ligand binding and in the absence of ligands. Due to these functional resemblances to vGPCRs, we highlight key features of ACKR1–4 here.

ACKR1 is the oldest known chemokine receptor, identified in 1950 as a blood group antigen, and was originally given the name Duffy (11). By binding more than 20 CXC and CC chemokines, it works as an extremely broad-spectrum receptor that serves as a chemokine sink in the blood through its high expression in erythrocytes. It is also expressed in the endothelial cells of small veins, where it internalizes chemokines and transports them to the apical cell surface to immobilize and expose them here (11).

ACKR2, formerly known as D6, shares the highest homology to CCRs and binds only inflammatory CC chemokines. It is widely expressed in barriers in the body and on various leukocyte subsets. It is constitutively internalized and recycled with only a minor fraction expressed at the cell surface (11). ACKR2’s main role is to regulate chemokine levels through internalization and subsequent lysosomal degradation. While the constitutive internalization is arrestin independent, the chemokine-scavenging role is arrestin dependent (16).

ACKR3 is most closely related to CXCR4, hence binding CXCL12, as well as the full agonist for CXCR3, CXCL11. It is widely expressed in hematopoietic and mesenchymal cells and in the nervous system. Via continuous internalization, it scavenges CXCL11 and −12 by lysosomal degradation, a process that seems to involve arrestin recruitment (17). Moreover, it also attenuates CXCR4 function by heterodimerization (18).

ACKR4 binds and internalizes multiple CC chemokines, yet with a different preference than ACKR2. Arrestin-dependent internalization controls chemokine concentrations necessary for the proper migration of CCR7- and CCR9-expressing cells (19) and attenuates CXCR3 via heterodimerization (20).

### 1.5. Herpesvirus Exploitation of G Protein–Coupled Receptors and Their Ligands

**Figure 3:**
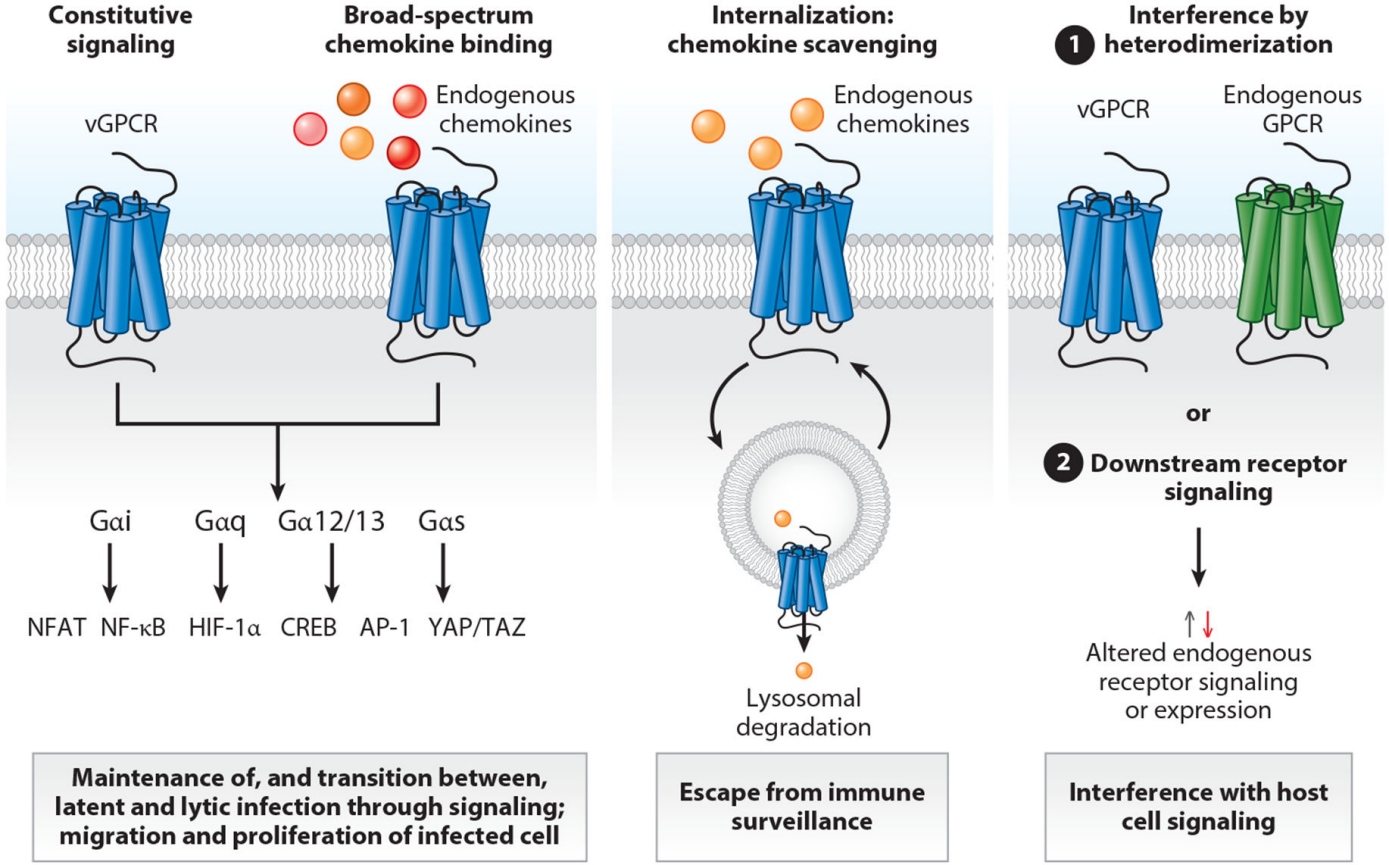
Shared properties of the vGPCRs. The vGPCRs share broad constitutive signaling through multiple pathways. They also bind multiple chemokines and, through internalization, clear them from the surroundings of the virus-infected cells. Through direct interaction and indirect crosstalk on signaling, they affect the function of endogenous and other viral receptors. Abbreviations: CREB, cAMP response element binding protein; GPCR, G protein–coupled receptor; HIF-1α, hypoxia-inducible factor 1α; NF-κB, nuclear factor kappa-light-chain-enhancer of activated B cells; NFAT, nuclear factor of activated T cells; vGPCR, viral G protein–coupled receptor. Figure adapted from images created with BioRender.com.

**Figure 4:**
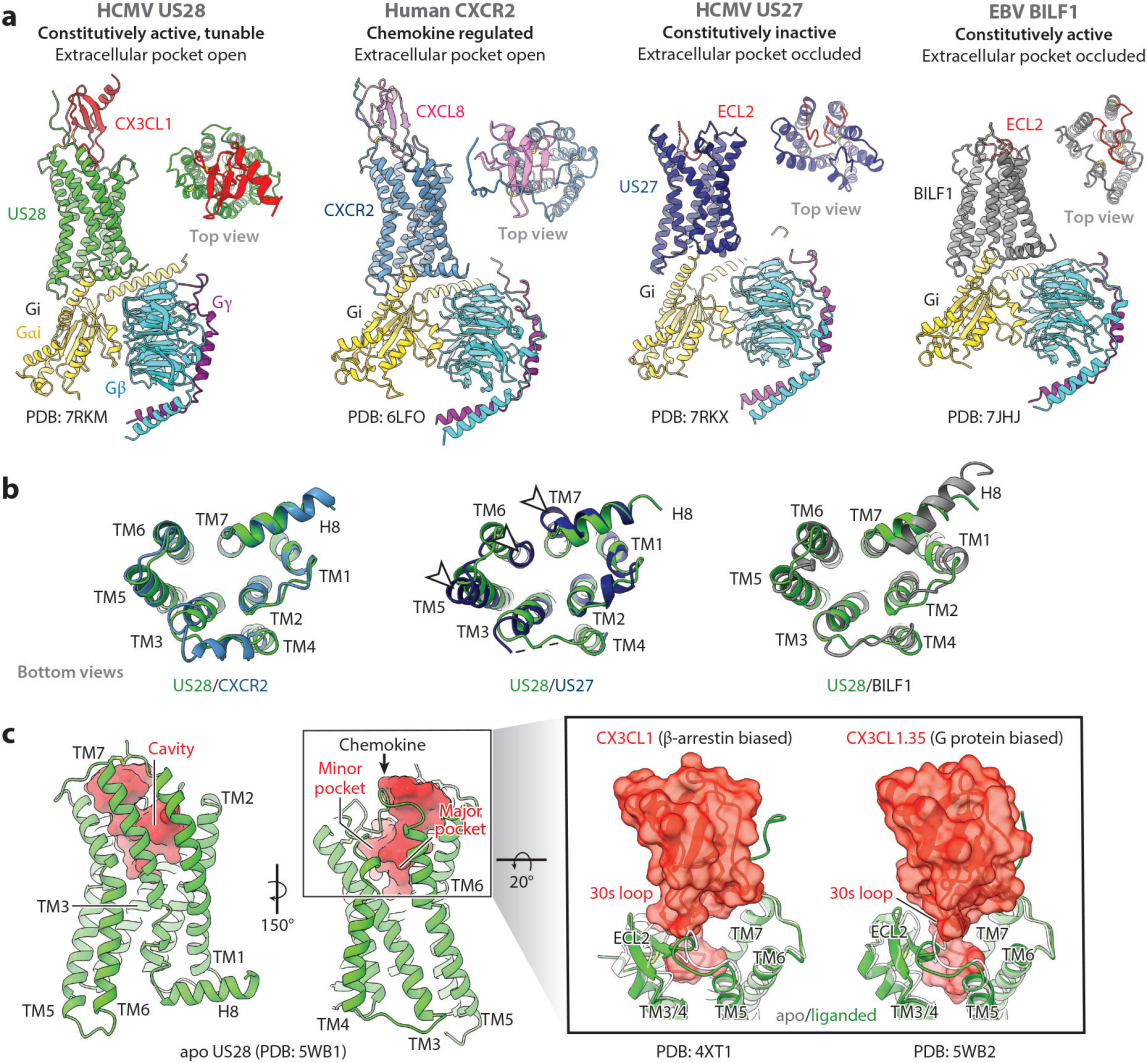
Structures of vGPCRs. (*a*) Cryo-EM structures of CX3CL1-US28-Gi (canonical-state), CXCL8-CXCR2-Gi, US27-Gi (canonical-like-state), and BILF1-Gi from the side view and the 90°-rotated top view. The models are colored in red (CX3CL1), green (US28), pink (CXCL8), blue (CXCR2), dark blue (US27), gray (BILF1), gold (Gα), cyan (Gβ), and purple (Gγ). ECL2s of BILF1 and US27 are highlighted in red. (*b*) Superimpositions of US28 model with CXCR2, US27, or BILF1 from the 90°-rotated bottom view from the side view in panel *a*, showing ligand-independent inactive and active conformation of US27 and BILF1, respectively. White arrowheads indicate relative TM positions between US27 and US28. (*c*) Crystal structures of apo US28, CX3CL1-US28, and CX3CL1.35-US28 assisted by a crystallization chaperone that is omitted for clarity. A ribbon model of apo US28 (*green*) is shown from two views with an extracellular cavity surface (*red*) on the left panels. The box indicates the region magnified on the right panels, showing binding modes between US28 (*green*) and CX3CL1s (*red*). For comparison, the apo US28 (*light gray*) is overlaid on the liganded US28 (*green*). Abbreviations: EBV, Epstein-Barr virus; ECL2, extracellular loop 2; HCMV, human cytomegalovirus; PDB, protein database; TM, transmembrane; vGPCR, viral G protein–coupled receptor. Figure prepared using UCSF ChimeraX (153).

Large double-stranded DNA viruses, such as herpes- and poxviruses, have acquired GPCRs, their ligands, or ligand-binding proteins to control and escape immune surveillance to promote spreading or control host cellular signaling for the benefit of their survival in the host (Figure 1). Recent research has uncovered major differences in molecular pharmacology and structure between the virus-encoded receptors and their closest endogenous homologs (Figures 3 and 4). Strikingly, their ligand-binding profiles are often broad spectrum and include chemokine ligands for several different receptors—even crossing the CC-CXC-XCL-CX3CL boundaries. Thus, through evolution, the viruses have optimized the captured chemokine receptors toward promiscuity rather than selectivity. Combined with the most often strong constitutive and ligand-induced receptor internalization, this allows the vGPCRs to function as chemokine scavengers and thereby prevent leukocyte recruitment to the virus-infected cells (21).

The signal transduction pathways of vGPCRs are also distinct from the endogenous GPCRs by being broad on the level of the G protein exploration and the downstream pathways (e.g., kinases, small GTPases, and transcription factors) (Figure 3). Notably, many vGPCRs display constitutive signaling through multiple pathways—a phenomenon that, indeed, distinguishes them from their endogenous homologs. In several cases, this robust constitutive activity has been linked to cellular transformation and oncogenicity of the viruses (21, 22). This difference in signaling from the endogenous counterparts is likely a reflection of the different biological properties for the virus-encoded receptors being important for several aspects of virus life cycle. These include cellular entry and survival, replication, dissemination, and to a minor extent cell migration, in contrast to the functions of endogenous chemokine receptors (migration, cellular activation, and differentiation).

Besides regulating the expression of endogenous chemokine and oxysterol receptors, the vGPCRs modulate the function of endogenous GPCRs by means of direct interaction through heterodimerization and by indirect impact on downstream signaling of these (Figure 3). These common denominators for vGPCRs are overviewed in Sections 2 and 3 focusing on vGPCRs identified in β-herpesviruses and γ-herpesviruses, respectively, and their druggability is briefly discussed in Section 4. The virus-encoded chemokines and chemokine-binding proteins are not the focus of this review, but given their central role in the way viruses manipulate and escape the immune system, they are briefly described in the following sections.

#### 1.5.1. Herpesvirus-encoded chemokines.

Viral chemokines have been identified in α-, β-, and γ-herpesviruses (Figure 1). Despite the conserved chemokine motifs, only approximately half of these have been matched with an endogenous chemokine receptor (23). The three KSHV-encoded CC chemokines, vCCL1–3 (vMIP1–3), are the most thoroughly characterized. While vCCL1 and −3 are narrow agonists for CCR8 (vCCL1) and CCR4 and XCR1 (vCCL3), vCCL2 acts as a broad-spectrum neutral antagonist that blocks various CC, CXC, XC, and CX3C receptors (CCR1, 2, 5, 10; CXCR4; CX3CR1; and XCR1), while activating CCR3 and CCR8 (24-27) (Figure 2). The receptor promiscuity of vCCL2 allows inhibition of natural killer (NK) and T helper 1 cell migration through antagonism and recruitment of Th2 cells through agonism, a property shared with vCCL1 and −3. HHV6 uses a similar approach by secreting U83A and -B (from HHV6A and -B, respectively), controlling cell migration toward the virus-infected cells through CCR activation (28, 29). HCMV is the only virus that encodes CXC chemokines in its genome. Of these, UL146 controls neutrophils and NK cell recruitment to infected cells with activation of CXCR1, −2, and −4, whereas UL147 remains an orphan (30).

#### 1.5.2. Herpesvirus-encoded chemokine-binding proteins.

Viral chemokine-binding proteins mostly occur in the genomes of poxviruses, but a few herpesviruses have also adapted this method to manipulate host chemokines, most often to inhibit their function but in rare cases to enhance it (31). The majority interfere with the common positively charged GAG-binding motifs of chemokines, a strategy that results in broad-spectrum chemokine scavenging, while some also interact with the GPCR interface of the ligands. Among the HHVs, HCMV encodes a chemokine-binding protein in the small, secreted glycoprotein pUL21.5 blocking CCL5 function (32), while HSV1/2 and VZV encode the glycoproteins gG and gC, respectively, that enhance chemokine function (31, 33).

## 2. β-HERPESVIRUS-ENCODED G PROTEIN–COUPLED RECEPTORS

All three human β-herpesviruses contain vGPCRs (Table 1). HCMV causes lifelong infection and is widely spread with a 50–100% seroprevalence depending on socioeconomic and geographical factors (6). The primary infection is usually asymptomatic but may cause mononucleosis-like symptoms. However, in immunosuppressed individuals, either as primary infection or upon reactivation from latency, this virus causes deadly infections—a common and severe complication in transplantation settings (6). Moreover, HCMV is the leading infectious cause of congenital infections. HHV6 and −7 are more widespread and cause exanthema subitum, a benign childhood disease, and are in rare cases linked to hepatitis. HHV7 has also been described as the causative agent in pityriasis rosea, a skin disease in adults, whereas HHV6, like many other viruses, is discussed as a potential player in multiple sclerosis (6).

### 2.1. Four G Protein–Coupled Receptors Are Found in the Human Cytomegalovirus Genome

Among the four vGPCRs found in HCMV, US27, US28, and UL33 show homology to chemokine receptors, whereas UL78 displays no homology to any endogenous receptor. US27 and US28 are unique for human and nonhuman primate cytomegalovirus (CMV), whereas UL33 and UL78 both are conserved among multiple mammalian CMVs (22).

#### 2.1.1. Ligand-binding profiles, signaling patterns, and receptor internalization.

US28 binds a variety of endogenous inflammatory CC chemokines belonging to the MIP and the MCP subclasses (CCL2–5 and −7) in addition to CX3CL1 (24, 34-36) (Figure 2). Intriguingly, it binds CC chemokines and CX3CL1 differently, as shown more than three decades ago. Simple ligand-binding competition assays revealed that iodinated CX3CL1 was displaced only by unlabeled CX3CL1 but not by CC chemokines, despite the comparable high-affinity binding of all CC chemokines in homologous competition settings (36). US28 signals through a broad range of downstream pathways, with strong signaling through Gαq, but is also capable of signaling through Gαi and Gα12/13, and downstream activation of multiple protein kinases, mitogen-activated protein (MAP) kinase, and transcription factors (37-42). Besides strong constitutive activity through these pathways, US28 is tuned by the CC chemokines acting as agonists and CX3CL1, in a context-dependent manner, as either partial or inverse agonist (34, 37, 41-45). US28 also recruits arrestins; however, these are not required for US28’s fast, constitutive, and clathrin-dependent internalization (46-48). While US27, UL33, and UL78 also internalize constitutively, no chemokine binding has been described, and little is known about their regulation and signaling (49, 50).

#### 2.1.2. Function in virus life cycle.

US28 mediates vascular smooth muscle cell migration in vitro in a Gα12-dependent manner, which has been suggested to impact HCMV infections in vivo (44, 45, 51, 52). Because of its high constitutive activity, US28 also induces cell transformation and carcinogenesis (53-56). The broad-spectrum chemokine binding and fast internalization suggest that US28 scavenges chemokines as a chemokine sink, thereby protecting the virus-infected cell from being recognized by leukocytes (36, 57, 58). This phenomenon has been used to target and kill lytic and latently HCMV-infected cells by designed selective US28-targeting chemokine-based fusion-toxin proteins (59-62). US28 plays a significant role in both the latent and lytic infection states. For instance, during lytic infection, it enhances cell-to-cell spread and/or maintenance of latency, in which both the constitutive activity and the broad chemokine binding are involved (43, 61, 63-68).

All four HCMV-encoded vGPCRs affect the function of endogenous chemokine receptors either directly through heterodimerization or indirectly by scavenging intracellular signaling components. In some cases, this prevents the functions of endogenous receptors (69, 70), whereas in other cases, an enhancement is observed (71-74).

#### 2.1.3. Structural knowledge.

The structures of US28 and US27 elucidate HCMV’s evolutional strategies to hijack host G protein signaling and scavenge chemokines (75-77). Recently, cryo-EM structures of human Gi-coupled CX3CL1-US28 and apo US27 were resolved, showing unique examples of host-pathogen protein-protein interactions (77) (Figure 4a). US28 and US27 were acquired by HCMV relatively recently and are restricted in human and primate CMVs (78). They have a clear human ortholog, CX3CR1, which is monogamously engaged with CX3CL1 for extracellular chemokine sensing and Gi for G protein signaling (79). Despite the shared ancestor, US27 has unknown chemokine and G protein partners, indicating it either completely altered or lost the chemokine-binding ability and Gi activity. Unlike US28, US27 lacks a chemokine-binding pocket that is plugged by extracellular loop 2 (ECL2) and the N-terminal region (Figure 4a). The intracellular end of TM6, a crucial indicator of the GPCRs’ activation state (10), is largely inwardly placed compared to US28 (Figure 4b), suggesting US27 is folded into a constitutively inactive conformation in a ligand-independent manner, while maintaining Gi binding without activation.

In complex with Gi, two distinct docking modes were observed for each US28 and US27 (77). One of the CX3CL1-US28-Gi modes resembles canonically active human GPCR-G protein complex structures (Figure 4a), thus classified as the canonical-state. The other, termed the orthocanonical-state, shows an entirely distinct GPCR-Gi docking mode with ~90° rotation and high tilt of Gi toward the membrane plane (77). The Gi tilt indicates that the complex can accommodate membrane curvatures of endosomal vesicles in addition to cell surfaces, proposing its Gi sink function analogous to chemokine sink (36, 57, 58). Similarly, US27-Gi adopts two distinct engagement modes with the Gi rotation: canonical-like-state and orthocanonical-like-state, both with weak GPCR-Gi interactions. Except for the canonical-like US27 complex, which flexibly anchors Gi only via the C-terminal tail of Gαi, the US28 complexes and the US27 complex bridge Gα and Gβ chains of nucleotide-free and GDP-bound Gi, respectively. This receptor-mediated stabilization of a Gi heterotrimer likely results in less efficient Gi signaling than the robust Gq signaling in vitro (38, 42, 77), which may redirect cellular G protein signaling to Gq bias that drives the proliferation of HCMV-infected cells.

The chemokine binding of US28 is also structurally well characterized with active-like-state crystal structures reported in complex with either CX3CL1 (N terminus: QHHGVTK) or its engineered version (CX3CL1.35, N terminus: VRPHINN), along with the apo form (75, 76) (Figure 4c). US28 recognizes both the β-arrestin-biased CX3CL1 and the G protein–biased CX3CL1.35 (76) similarly but with distinct binding mode from CXCL8-CXCR2 (Figure 4a) (80) and vCCL2-CXCR4 (81) as a result of a significant chemokine rotation. Still, they all follow a canonical two-step binding via Site 1 and 2 interfaces with Site 1.5 (75, 76, 81). The notable differences between the two CX3CL1 variants are their 30s-loop and peptide engagement modes to US28 (right side of Figure 4c). In the wild-type complex, the 30s loop touches down onto US28’s ECL2, whereas CX3CL1.35 makes a restricted ECL2 contact with the 30s loop but shows more substantial interactions between the chemokine domain and US28’s TM6/7. Concerning the N-terminal part, the wild-type peptide reaches only the minor pocket, while the engineered peptide projects into both the minor and major pockets pushing TM5/6. Despite the substantial differences in the N-terminal sequences, ligand recognition modes, and signaling properties between the two ligands, they maintain high affinities toward US28, probing the plasticity in extracellular ligand recognition underlying the promiscuous chemokine sink function of US28 (36, 57, 58).

While the structural studies have advanced our understanding of the chemokine- and Gi-binding mechanisms by US27 and US28, several major structures remain elusive: (*a*) US28 bound to CC chemokines, (*b*) an active-state US28-Gq complex and a US28-G12/13 complex, (*c*) a vGPCR-β-arrestin complex, (*d*) homo/hetero vGPCR dimers, and (*e*) the other two vGPCRs encoded by HCMV. Therefore, further exploration will be necessary to describe the structural mechanisms behind their biological functions.

### 2.2. Human Herpesvirus 6– and 7–Encoded U12 and U51

Two different receptors, U12 and U51, are found in both HHV6 and −7. Of these, U12 resembles the UL33 subclass of vGPCRs, whereas U51 shows closest homology to the UL78 subclass (22).

#### 2.2.1. Ligand-binding profiles and signaling patterns.

U12 and U51 from HHV6 and −7 bind CC chemokines (Figure 2). The two receptors from HHV7 bind the same four chemokines, CCL17, −19, −21, and −22, thus interfering with chemokines recognized by CCR4 and −7 (82, 83). In contrast, U51 from HHV6 binds an extensive set of CC chemokines, CX3CL1, and XCL1, while U12 binds a few inflammatory CC chemokines (CCL2–5) (84, 85) (Figure 2).

#### 2.2.2. Function in virus life cycle.

Only little is known about these receptors’ role in the virus life cycle. The broad chemokine binding and signaling are well established using isolated receptor expression systems but not in the context of the virus-infected cells. A study suggested that U51 signaling regulates HHV6 replication (86), and that the overall broad chemokine binding is likely to influence leukocyte migration. However, this remains to be finally established.

## 3. γ-HERPESVIRUS-ENCODED G PROTEIN–COUPLED RECEPTORS

The two different subfamilies of the γ-herpesviruses each contain one vGPCR. EBV encodes BILF1, which is highly conserved among mammalian lymphocryptoviruses (87). In KSHV the receptor is in open reading frame 74 (ORF74) and is also conserved among animal γ-herpesviruses, such as the nonhuman primate herpesvirus saimiri (HVS), the equine herpesvirus 2 (EHV2), and the murine γ-HV68 (MHV68) (88-91).

### 3.1. Epstein-Barr Virus–Encoded BILF1

EBV is found in more than 90% of adults worldwide (6). Primary infection generally occurs in childhood or adolescence through contact with bodily fluids such as saliva of an infected person, after which EBV enters host epithelial cells (6). This infection can cause mononucleosis, also called kissing disease, which presents as a typically self-containing and mild fever. After this phase of lytic replication, EBV enters its latent cycle in naïve B cells and ultimately resting memory B cells. Here, the gene expression is limited to a number of (or no) latent genes depending on the exact latency program (0, I, II, III), in order to evade the immune system and persist in the host (6). EBV was initially identified in Burkitt’s lymphoma tissue (92) but is now associated with multiple cancers (93). While predominantly being involved in various lymphomas in immunocompetent and immunosuppressed persons, EBV-associated carcinomas, such as nasopharyngeal carcinoma among others, are the cause of the majority of EBV-associated malignancies (93).

#### 3.1.1. Signaling profiles and receptor recycling and internalization.

BILF1 is a heavily glycosylated vGPCR expressed in EBV-infected cells (94). It signals through G proteins, more specifically through the inhibitory subtype Gαi (94, 95). The DRY motif (Asp-Arg-Tyr) in the bottom of TM3 of class A GPCR is moderately conserved as EKT (Glu-Lys-Thr) in BILF1. This motif is essential for Gi coupling by BILF1 as implicated by the signaling-incompetent K122A mutant (EKT changed to EAT) (96). Moreover, BILF1’s constitutive signaling is known to activate the transcription factors nuclear factor kappa-light-chain-enhancer of activated B cells (NF-κB), nuclear factor of activated T cells (NFAT), and modulate cAMP-response element (CRE) (87). It has no known ligands, and its constitutive signaling is believed to stem from several evolved structural peculiarities (see Section 3.1.4) (97). Furthermore, as recently described, this vGPCR is constitutively internalized and recycled back to the cell surface (48).

Connected to constitutive signaling, BILF1 is a known EBV oncogene (96). Here, the BILF1 wild-type and the signaling-incompetent K122A mutant were analyzed in vitro using NIH-3T3 cells. While the wild-type BILF1 induced foci formation, the K122A mutant did not. Furthermore, this mutant could not disrupt the contact inhibition of NIH-3T3 cells, drive cell transformation, and increase vascular endothelial growth factor (VEGF) secretion. However, the K122A mutant still led to tumor formation in a mouse xenograft model of bilaterally subcutaneously injected nude mice. Despite developing tumors in only 60% of mice (and 40% of injection sites)—compared to 100% of mice (and 87.5% of injection sites) for mice injected with BILF1 wild-type NIH-3T3 cells—these results indicate alternative pathways in BILF1-induced tumorigenesis.

#### 3.1.2. Interplay with other G protein–coupled receptors.

BILF1 interacts with endogenous GPCRs and potentially modulates certain signaling pathways. Among the discovered heterodimers of BILF1 and CCR9, CCR10, CXCR3, or CXCR4 (98), the interaction of BILF1 and CXCR4 has been studied in greatest detail (99). CXCL12 binding to CXCR4 was attenuated in a BILF1 signaling–dependent manner without drastically reducing CXCR4 expression. As BILF1 also formed heterodimers with the histamine receptor H4R, but did not interfere with histamine binding to its receptor, it was concluded that the interference of CXCL12-CXCR4 signaling was related to constitutive G protein scavenging by BILF1. Downstream CRE signaling was impaired for both CXCR4 and H4R, also suggesting a G protein scavenger function of BILF1.

#### 3.1.3. Function in virus life cycle.

Due to the constant pressure of the host immune system to clear the virus, EBV has evolved a multitude of mechanisms that allow it to evade eradication from the host. BILF1 plays a significant role in EBV immune evasion with multiple strategies that might also be linked to carcinogenesis. First, it downregulates the major histocompatibility complex (MHC)-I receptor surface expression to escape surveillance by cytotoxic T cells (CTLs) (100). This occurs through (*a*) the internalization and lysosomal degradation of surface MHC-I receptors and (*b*) the diversion of newly synthesized MHC-I receptors from the normal exocytic pathway. While early research suggested BILF1-Gi signaling independency (101), later studies concluded that the endocytic depletion of MHC-I is linked to BILF1 signaling and the interaction of its intracellular C-terminal tail with MHC-I (102). Moreover, a significant reduction of the MHC-I subtypes HLA-A, HLA-B, and HLA-E was observed, in contrast to the barely influenced subtype HLA-C (102). As HLA-A and HLA-B are responsible for CTL responses while NK cell responses are mediated through HLA-C, this MHC-I subtype bias could lead to effective evasion of EBV from CTLs (102). Additional studies on conserved amino acids in the BILF1 extracellular regions also propose their roles in MHC-I downregulation (103). Another antiviral host cell pathway meant to limit viral protein synthesis through the protein kinase R (PKR)-dependent phosphorylation of the eukaryotic initiation factor, and induce apoptosis of infected cells, is inhibited by BILF1 through an inhibition of the phosphorylation of PKR (95).

#### 3.1.4. Structural knowledge.

BILF1 does not show a high similarity to a specific human GPCR, yet it has been considered an ortholog of CXCRs (95). The structure of the EBV BILF1-human Gi complex unveiled marked differences from chemokine receptors, where the extracellular chemokine-binding pocket is occluded by ECL2 (97) (Figure 4a). This feature is shared with US27, proposing a common viral strategy to acquire a ligand-independent GPCR. In contrast to the inactive US27, BILF1 modified its class A GPCR microswitches to ensure its constitutive activity (87, 97). For example, BILF1 does not possess the ionic lock that stabilizes the inactive GPCR conformation by a salt bridge between TM3 and TM6 and the sodium-binding pocket, which is an established negative allosteric modulation site with signature residues (10). Furthermore, it lost a critical proline residue on TM7, which works as a conformational switch to facilitate G protein binding (Figure 4b), as observed in other constitutive active GPCRs (104). With the stable active conformation, BILF1 binds Gi similarly to endogenous GPCR-Gi complexes, hijacking the Gi signaling in a way opposite from HCMV GPCRs, likely exhausting the Gi pool in the infected cells (99).

Based on the lack of the extracellular chemokine-binding pocket and unsuccessful identification of chemokine ligands (95), it is clear that BILF1 is not a typical chemokine receptor. Furthermore, BILF1 had an open pocket within the transmembrane region, between TM6 and TM7, with a shimmed unidentified molecule (97). Together with the ECL2 lid, these features imply that BILF1 may be a lipid GPCR. However, as the identity and function of this molecule are unclear, BILF1 still remains an orphan vGPCR.

### 3.2. Kaposi’s Sarcoma-Associated Herpesvirus–Encoded Open Reading Frame 74

KSHV is the established agent of the endothelial tumor called Kaposi’s sarcoma (KS) and is associated with two additional human malignancies: primary effusion lymphoma and the lymphopro-liferative disorder known as multicentric Castleman’s disease (105, 106). KSHV is concentrated in specific areas such as sub-Saharan Africa (seroprevalence: 30–80%) and Mediterranean populations (10–20%) and with only low prevalence in northern Europe and North America (1–5%); however, in the immunosuppressed, its prevalence is markedly increased (6). The primary infection is usually asymptomatic but may result in a syndrome consisting of fever, rash, lymphadenopathy, and bone marrow failure, for instance, in post-transplant patients (6). Like other herpesviruses, it enters latency, where infected cells contain multiple copies of circular KSHV genome maintained by the latency-associated nuclear antigen (106). Many signals may provoke lytic replication, but spontaneous reactivation also occurs, and the switch between latent and lytic replication is known as the ORF50 replication transactivator. As discussed below, ORF50 expression is controlled by ORF74, thereby switching from latent to lytic replication (107).

#### 3.2.1. Ligand-binding profiles, signaling, and receptor internalization.

ORF74 is conserved among many γ2-herpesviruses, of which the HVS homolog was the first to be characterized in 1993 (88). Later, it was cloned from KSHV-positive KS lesions (108), EHV2 (109), MHV68 (91), and rhesus rhadinovirus (110). All ORF74s share high homology to CXCR2 and are consistently activated by the ELR CXC chemokines acting as CXCR2 agonists (88-90, 111). For KSHV ORF74, CXCL1–3 are potent agonists, while CXCL5, −7, and −8 are partial agonists through Gq signaling (112-114). ORF74 also binds non-ELR CXC chemokines with high affinity. However, while CXCL10 and CXCL12 are agonists for the endogenous CXCR3 and CXCR4, respectively, they act as inverse agonists on ORF74 to inhibit its high constitutive activities (113-115). In addition, vCCL2 binds to ORF74 as an inverse agonist (115).

ORF74 displays high constitutive activity primarily through Gαq but also via Gαi and Gα12/13 (42, 113, 116-118). Downstream of the G proteins, it activates a multitude of signaling pathways, resulting in the activation of protein kinases and MAP kinases, and at the transcriptional level of hypoxia-inducible factor 1α, CRE, NFAT, NF-κB, and serum response element (42, 119-124). ORF74 also activates YAP/TAZ, two oncoproteins inhibited by the Hippo suppressor pathway in a simian virus 40 (SV40) early promoter–immortalized murine endothelial cell line, in KS-like mouse tumors, and in clinical human KS specimens (125). The high constitutive activity of ORF74 is directly linked to its oncogenic properties, as shown by ORF74-expressing cells, transplanted under the skin of nude mice, resulting in highly vascularized tumors via VEGF secretion (116). Similar results were obtained in another transgenic model expressing ORF74 under SV40 (126). As a more relevant disease model, the CD2 promoter-driven ORF74 expression is sufficient for developing KS-like lesions (127). Notably, using this model, a signaling-deficient ORF74 variant did not induce tumor formation, and another variant devoid of agonist binding resulted in a lower degree of tumor formation, suggesting that both the ligand-dependent and -independent ORF74 signaling play significant roles in KSHV-associated tumorigenesis (128, 129).

ORF74 internalizes upon binding of CXCL1 and −8 via the β-arrestin-dependent pathway and subsequently traffics back to the cell surface via early endosomes or accumulates in late endosomes (130).

#### 3.2.2. Function in virus life cycle.

ORF74 is expressed in the lytic phase, and as alluded to above, it has a strong oncogenic potential. However, studies of virus-host interactions and their influence on pathogenesis in the host have been hampered due to the inability to establish an infection in cell cultures and the lack of appropriate small animal models to study this and other herpesviruses. As the natural γ-herpesvirus pathogen of murid rodents (MHV68) encodes an ORF74 receptor with overlapping ligand-binding profiles to ORF74 (111), it provides a relevant model for such studies. It shares both genomic and pathologic similarities with KSHV and EBV (131-133) and readily infects mice where it establishes a chronic infection allowing both lytic and latent infections to be studied (134). From such studies in mice, MHV68 ORF74 was found dispensable for an acute infection but necessary for reactivation from latency (135, 136). A similar role was described in vitro for ORF74, where the induction of the ORF50 promoter was dependent on the transcription factors Sp1 and Sp3, involving signaling via Gα12 and Gαq (107). Two other studies corroborated a significant role of ORF74 for the control of ORF50 activity, specifically in the switch from latency to lytic replication through its G protein signaling (137, 138).

#### 3.2.3. Structure-function knowledge.

No structure is currently available; however, as mentioned above, ORF74 is considered a CXCR2 homolog with a sequence identity and similarity of ~27% and ~67%, respectively. With the overlapped chemokine- and G protein–binding profile, the signaling ORF74 complex might resemble the CXCL8-CXCR2-Gi structure reported recently (80) (Figure 4a). Besides, the previous mutagenesis studies have identified critical regions for receptor activity, expression, and ligand binding (118, 128-130, 139-142). One such region is the conserved DRY motif at the bottom of TM3, which is VRY in ORF74 (128, 140). As in most chemokine receptors, the N-terminal region is needed for chemokine binding (128, 143), and a positively charged motif in the extracellular top of TM5 appears essential for agonist binding (128). Furthermore, at the intracellular interface, the hydrogen-bonding network formed between TM2, TM7, and helix 8 has been proposed to enhance chemokine binding allosterically (141). Other motifs in the C terminus have been identified as important, such as the AP2 consensus binding site YGLF that directs ORF74 localization between the plasma membrane and clathrin-coated vesicles (142).

## 4. CONCLUSION

GPCRs are involved in diverse aspects of cell signaling in the body and thus are relevant in human health, as exemplified by 34% of all Food and Drug Administration–approved drugs that target GPCRs (144). Hence, GPCRs are druggable proteins, potentially exploited for novel drugs for viral instead of endogenous GPCRs (21). The prime example is US28, although no drug candidate has yet reached the market. Several small-molecule ligands have shown promise in attenuating oncogenic US28 signaling and chemokine binding (145-147). Besides these, single-domain antibodies are under investigation, suppressing the growth of US28-expressing glioblastoma cells through attenuation of US28 signaling and viral reactivation (148-151). Another strategy is to exploit US28’s constitutive internalization for toxin uptake (59-61). A CX3CL1 variant that selectively binds to US28 but not to endogenous CX3CR1 was fused to a *Pseudomonas* endotoxin A moiety to selectively kill patient-derived HCMV-infected cells in vitro even during viral latency (61) and ex vivo in lung perfusion systems, underlying ex vivo HCMV elimination in solid organ transplantations (62). ORF74 and BILF1 have been topics of therapeutic intervention through metal-ion site engineering for exploring potentials of designing therapeutics to shut down their signaling involved with virus life cycle and virus-mediated oncogenesis (103, 113).

In conclusion, vGPCRs have substantial structural and functional differences from typical class A GPCRs but often share similarities to endogenous chemokine receptors, including atypical chemokine receptors. The receptors possess various roles in the viral life cycle, such as establishing latency and suppressing host immunity during the acute lytic phase, and show oncogenic activities. Thus, they can be druggable by various modes of action, which hold great promise to treat diseases associated with human herpesviruses.

## Figures and Tables

**Table 1:** Overview of human herpesviruses and their content of vGPCRs and viral chemokines

Herpesvirus	Subfamily	Primary disease(s)	Disease reactivation	Latency/ latencies	Viral receptors	Viral chemokines
HSV1/HHV1	*Alphaherpesvirinae*: simplexvirus	Often asymptomatic Oral herpes (cold sores) Eczema herpiticum	Oral herpes	Trigeminal ganglia	NA	NA
HSV2/HHV2	*Alphaherpesvirinae*: simplexvirus	Often asymptomatic Genital herpes Eczema herpiticum	Genital herpes	Dorsal root ganglia	NA	NA
VZV/HHV3	*Alphaherpesvirinae*: varicellovirus	Chicken pox	Zoster (shingles)	Trigeminal and dorsal root ganglia	NA	NA
EBV/HHV4	*Gammaherpesvirinae*: lymphocryptovirus	Infectious mononucleosis	Often asymptomatic but potentially deadly	B cells and epithelial cells	BILF1	NA
HCMV/HHV5	*Betaherpesvirinae*: cytomegalovirus	Often asymptomatic; rarely mononucleosis-like	Often asymptomatic but potentially deadly	CD34+ myeloid progenitors and CD14+ monocytes	US27, US28, UL33, UL78	UL146, UL147
HHV6A/B	*Betaherpesvirinae*: roseolovirus	Exanthema subitum	Usually asymptomatic; rarely hepatitis	Monocytes and CD34+ stem cells	U12, U51	U83A/B
HHV7	*Betaherpesvirinae*: roseolovirus	Exanthema subitum Pityriasis rosea	Usually asymptomatic; rarely hepatitis	CD4+ T cells	U12, U51	NA
KSHV/HHV8	*Gammaherpesvirinae*: rhadinovirus	Usually asymptomatic; rarely fever and rash	Usually asymptomatic, but potentially oncogenic	B cells and endothelial cells	ORF74	vCCL1, −2, −3

From left, the family and subfamily of the human herpesviruses are listed along with the diseases from primary infections and virus reactivation from latency and the main host cells during latency. The last two columns describe the viral GPCRs from each herpesvirus and the viral chemokines. Abbreviations: EBV, Epstein-Barr virus; GPCR, G protein–coupled receptor; HCMV, human cytomegalovirus; HHV, human herpesvirus; HSV, herpes simplex virus; KSHV, Kaposi’s sarcoma-associated herpesvirus; NA, not available; ORF, open reading frame; vGPCR, viral G protein–coupled receptor; VZV, varicella-zoster virus. Data obtained from References 6 and 7.
